# Validation of the Generalized Anxiety Disorder-7 in patients with COPD: a cross-sectional study

**DOI:** 10.1186/s12888-023-05072-5

**Published:** 2023-08-15

**Authors:** Meishan Liu, Dong Wang, Jiexin Fang, Yuhan Chang, Yongdong Hu, Kewu Huang

**Affiliations:** 1 Department of Respiratory and Critical Care Medicine, Beijing Institute of Respiratory Medicine and Beijing Chao-Yang Hospital, Capital Medical University, No 8, Gongti South Road, 100020 Chaoyang District, Beijing, PR China; 2 Department of Clinical Psychology, Beijing Chao-Yang Hospital, Capital Medical University, No 8, Gongti South Road, 100020 Chaoyang District, Beijing, PR China

**Keywords:** Chronic obstructive pulmonary disease, Generalized anxiety disorder, GAD-7, Screening, Validation

## Abstract

**Background:**

Patients with chronic obstructive pulmonary disease (COPD) often have comorbid generalized anxiety disorder (GAD), which requires early screening in respiratory clinics. The Generalized Anxiety Disorder-7 (GAD-7) questionnaire is a brief and commonly used screening tool for GAD but has not been validated among patients with COPD in China.

**Methods:**

Stable patients with COPD from a cross-sectional observational study were assessed using the GAD-7 questionnaire and then assessed by a senior psychiatrist to confirm a diagnosis of GAD according to the criteria of the Diagnostic and Statistical Manual of Mental Disorders, Fifth Edition. Demographic characteristics, spirometry, and patient-reported outcomes were collected. Cronbach’s α coefficient was calculated, and receiver operating curve (ROC) analysis was performed to validate the GAD-7.

**Results:**

A total of 226 patients with COPD were enrolled, and 50 (22.1%) of these patients were diagnosed with GAD. The Cronbach’s α coefficient for the GAD-7 was 0.869, which indicated good internal consistency. ROC curve analysis showed that the GAD-7 had an area under the curve (AUC) value of 0.829 (95% CI: 0.774–0.876) for identifying GAD. The optimal cut-off score was ≥ 4, with a sensitivity of 66.0% and a specificity of 89.2%. Higher GAD-7 scores were significantly associated with health-related quality of life and the symptom burden of COPD. The discriminatory power of GAD-7 did not differ statistically when stratified by COPD severity.

**Conclusions:**

The GAD-7 was shown to be a reliable and valid screening tool for patients with COPD in China, and its screening performance for GAD was not influenced by disease severity.

## Background

Chronic obstructive pulmonary disease (COPD) is a worldwide health concern, resulting in 3.3 million deaths and 74.4 million disability-adjusted life-years globally [1]. COPD is characterized by persistent airflow limitation and is always complicated by various systemic comorbidities. Anxiety is one of the most common comorbidities in COPD but is often undiagnosed. The prevalence of clinical anxiety ranges from 10% to 55% among inpatients with COPD and 13% to 46% among outpatients with COPD [2]. Comorbid anxiety is associated with an increased risk of COPD exacerbation [3–5], poorer health-related quality of life [6–8], more frequent hospitalization [9], longer hospital stays [10, 11], higher mortality rates, and increased healthcare utilization [11–13]. Therefore, early screening for anxiety disorders in patients with COPD is highly warranted.

Generalized anxiety disorder (GAD) is the most prevalent type of anxiety disorder in patients with COPD, with the major symptoms of excessive and uncontrollable worries [14]. It is difficult to diagnose GAD in patients with COPD because some somatic symptoms, such as fatigue and sleep disturbances, may be confused with symptoms that originate from COPD. In addition, most patients with COPD are elderly adults who are often unwilling to talk about their mental health and generally report fewer psychological symptoms [15].

The Generalized Anxiety Disorder-7 (GAD-7) questionnaire is one of the most commonly used screening tools for GAD which does not contain somatic symptoms. This questionnaire has been adapted into a Mandarin Chinese version and was validated in general hospital outpatients in China [16]. However, the Chinese version of the GAD-7 has not been validated among patients with COPD. A previous study in the United States found that the area under the curve (AUC) for the GAD-7 was 0.78, with a sensitivity of 77% and a specificity of 77% in patients with COPD [17], but this study only used the cut-off score recommended for the primary care population (GAD-7 ≥ 5) [18] without identifying the best cut-off score among patients with COPD, and the gold standard in the study was based on the Mini International Neuropsychiatric Interview (MINI) performed by trained coordinators but not experienced psychiatrists. No previous reports have focused on determining the best cut-off score for the GAD-7 among patients with COPD. The present study aimed to validate the GAD-7 questionnaire in patients with COPD in China and investigate the optimum cut-off score among these patients.

## Methods

### Study design and population

The Cohort Study for COPD in China (COMFORT study) is an ongoing multicenter prospective observational study that was initiated in June 2016 to investigate the clinical characteristics of patients with COPD in China. More details can be found at http://www.chinacopd.com/#/hot (ClinicalTrials.gov ID: NCT03044847). Patients with COPD from the COMFORT study at Beijing Chao-Yang Hospital were consecutively recruited in this cross-sectional survey if they consented to a psychiatric interview during routine outpatient visits. The inclusion criteria were as follows: 1) age 40 years or older; 2) diagnosed with COPD according to respiratory symptoms, risk factors, and a post-bronchodilator forced expiratory volume in 1 s/ forced vital capacity (FEV_1_/FVC) less than 0.70. The exclusion criteria were: 1) experienced acute exacerbations of COPD in the previous 30 days; 2) had other respiratory diseases with massive lung tissue destruction (such as severe tuberculosis or bronchiectasis); 3) had serious psychiatric disorders, such as severe cognitive impairment, intellectual disability, and psychotic disorders that interfered with the ability to understand the questionnaire and cooperate with the study. The study protocol was approved by the Ethics Committee of Beijing Chao-Yang Hospital (No.2021-KE-609), and the study was in accordance with the Declaration of Helsinki. All participants provided written informed consent.

### Data collection

Age, sex, smoking history, annual household income, education level, medications, comorbidities, and exacerbation history over the prior 12 months were collected based on patient reports. Exacerbations of COPD were defined as worsening of respiratory symptoms in fewer than 14 days that resulted in a change of at least one of these medications, namely antibiotics, corticosteroids, and/or bronchodilators, or necessitated a visit to the emergency room or hospitalization [19]. Patient-reported outcomes included assessments of dyspnea (modified Medical Research Council [mMRC] dyspnea scale); symptom burden (COPD Assessment Test [CAT]); and quality of life (St. George’s Respiratory Questionnaire [SGRQ]). Spirometry was performed using a Jaeger Masterscreen spirometer (Viasys Healthcare, Höchberg, Germany) before and after administration of an inhaled bronchodilator (salbutamol, 400 μg) according to the American Thoracic Society and European Respiratory Society guidelines [20]. Global Initiative for Chronic Obstructive Lung Disease (GOLD) stages and GOLD groups were categorized according to the updated 2023 GOLD report [19].

### Measurements

The GAD-7 is a self-reported questionnaire that consists of seven items about how often individuals have been bothered by several anxiety-related symptoms over the prior two weeks [18]. Each item was rated on a 4-point Likert scale as 0 (not at all), 1 (several days), 2 (more than half the days), and 3 (nearly every day), with total scores ranging from 0 to 21. We used the Chinese version of the GAD-7, which was validated in a previous study [16].

In the present study, a clinical diagnosis of GAD by a senior psychiatrist from Beijing Chao-Yang Hospital based on the Diagnostic and Statistical Manual of Mental Disorders, Fifth Edition (DSM-V) [21] was used as the gold standard. Each participant first received the GAD-7 questionnaire and was then assessed by the psychiatrist to confirm a diagnosis of GAD on the same day. The psychiatrist was blinded to the results of the GAD-7 to avoid bias.

### Statistical analysis

All analyses were performed using SPSS version 23.0 (IBM, Armonk, NY, USA) and MedCalc version 20.0 (MedCalc Software, Ostend, Belgium). *P*-values were two-sided, and *P*-values < 0.05 were considered statistically significant. Normally distributed data, determined using the Kolmogorov–Smirnov test or Q–Q plots, are presented as means and standard deviations; non-normally distributed data are presented as medians and interquartile ranges. Qualitative variables are presented as absolute and relative frequencies. The differences between patients with COPD with and without GAD were analyzed using the Student’s t-test or the Mann–Whitney U test for normally or non-normally distributed quantitative data, respectively. The Chi-squared test or Fisher’s exact test were used for categorical variables. The internal consistency of the GAD-7 was measured using Cronbach’s α coefficient. Receiver operating characteristic (ROC) curves were drawn to analyze the discriminatory power of the GAD-7. Operating characteristics, including the sensitivity, specificity, Youden index, positive predictive value (PPV), negative predictive value (NPV), positive likelihood ratio (+ LR) and negative likelihood ratio (-LR) were established at various cut-off scores of the GAD-7, with the optimum cut-off score chosen based on the largest Youden index. Correlation analyses of GAD-7 scores with demographics and clinical factors were performed using Spearman’s correlation. GAD-7 scores classified by age, sex, smoking status, education level, annual household income, COPD medication use, acute exacerbation history, GOLD stage, and GOLD group were compared using the Mann–Whitney U test or the non-parametric Kruskal–Wallis test. Comparisons of AUC values between different subgroups were performed using the Delong test [22].

## Results

### Sample characteristics

A total of 226 patients with COPD were enrolled in this study; of these patients, 50 (22.1%) were diagnosed with GAD. The demographic and clinical characteristics of the patients stratified by GAD diagnosis are shown in Table 1. The mean age of the participants was 67.5 ± 7.3 years and 88.5% were men. Over half of the participants (55.8%) were classified as GOLD group A, 28.3% as GOLD group B, and 15.9% as GOLD group E. Patients with GAD had significantly higher CAT scores, higher mMRC dyspnea scores, higher SGRQ scores and reported more exacerbations in the prior year than patients without GAD. The mean GAD-7 score was significantly higher in the GAD group compared with the no-GAD group (5.0 [1.8–8.0] vs. 0.0 [0.0–2.0], *P* < 0.001).

**Table 1:** Demographic and clinical characteristics of patients with COPD with or without GAD

Characteristics	Total (n = 226)	GAD (n = 50)	No GAD (n = 176)	*P* value
Age, years (mean, SD)	67.5 ± 7.3	67.6 ± 7.1	67.5 ± 7.4	0.886
Men (n, %)	200 (88.5)	46 (92.0)	154 (87.5)	0.379
BMI, kg/m^2^ (mean, SD)	25.1 ± 3.5	25.3 ± 3.4	25.0 ± 3.5	0.601
Smoking status (n, %)				
Non-smoker	32 (14.2)	8 (16.0)	24 (13.6)	0.912
Current smoker	73 (32.3)	16 (32.0)	57 (32.4)	
Former smoker	121 (53.5)	26 (52.0)	95 (54.0)	
Pack-years history (median, IQR)	40.0 (25.5–51.0)	41.0 (33.8–54.5)	38.0 (23.9–50.0)	0.117
Education level (n, %)				
Middle school or below	119 (52.7)	30 (60.0)	89 (50.6)	0.238
High school or above	107 (47.3)	20 (40.0)	87 (49.4)	
Annual household income, yuan (median, IQR)	87,000 (49,500–120000)	82,000 (47,500–120000)	90,000 (48,500–120000)	0.874
Comorbidities				
Asthma	47 (20.8)	13 (26.0)	34 (19.3)	0.304
Sleep apnea	13 (5.8)	5 (10.0)	8 (4.5)	0.264
Hypertension	68 (30.1)	16 (32.0)	52 (29.5)	0.738
Coronary artery disease	37 (16.4)	12 (24.0)	25 (14.2)	0.099
Gastroesophageal reflux	15 (6.6)	5 (10.0)	10 (5.7)	0.447
Diabetes mellitus	28 (12.4)	9 (18.0)	19 (10.8)	0.172
Exacerbations in the prior year (n, %)				
≥ 1	49 (21.7)	17 (66.0)	32 (18.2)	**0.017**
0	177 (78.3)	33 (34.0)	144 (81.8)	
Lung function (post-BD)				
FEV_1_, L (mean, SD)	1.78 ± 0.64	1.72 ± 0.71	1.79 ± 0.63	0.509
FEV_1_% predicted (mean, SD)	66.4 ± 22.2	62.9 ± 23.8	67.4 ± 21.7	0.204
GOLD stage (n, %)				
I–II	169 (74.8)	34 (68.0)	135 (76.7)	0.211
III–IV	57 (25.2)	16 (32.0)	41 (23.3)	
GOLD group (n, %)				
A	126 (55.8)	20 (40.0)	106 (60.2)	**0.033**
B	64 (28.3)	18 (36.0)	46 (26.1)	
E	36 (15.9)	12 (24.0)	24 (13.6)	
Medication for COPD (n, %)				
SABA	5 (2.2)	1 (2.0)	4 (2.3)	1.000
LAMA	23 (10.2)	6 (12.0)	17 (9.7)	0.629
LABA/LAMA	15 (6.6)	4 (8.0)	11 (6.3)	0.907
ICS/LABA	66 (29.2)	18 (36.0)	48 (27.3)	0.231
ICS/LABA + LAMA	59 (26.1)	16 (32.0)	43 (24.4)	0.282
mMRC (median, IQR)	1.0 (0.0–2.0)	2.0 (1.0–3.0)	1.0 (0.0–2.0)	** < 0.001**
CAT (median, IQR)	9.0 (5.0–15.0)	14.5 (8.0–21.0)	8.0 (4.0–13.0)	** < 0.001**
SGRQ (median, IQR)				
symptoms	33.1 (13.7–50.3)	46.2 (31.2–58.4)	26.9 (10.8–45.9)	** < 0.001**
activity	35.1 (17.3–53.3)	47.7 (23.3–72.8)	29.3 (17.1–42.1)	** < 0.001**
impacts	12.6 (5.2–25.9)	27.8 (7.6–42.3)	12.1 (5.0–18.5)	** < 0.001**
total	22.4 (12.1–36.4)	36.0 (17.8–52.2)	20.5 (10.8–31.8)	** < 0.001**
GAD-7 score (median, IQR)	1.0 (0.0–3.0)	5.0 (1.8–8.0)	0.0 (0.0–2.0)	** < 0.001**

Data are presented as mean ± SD, median (IQR), or n (%). Significant values are presented in bold

*BD* bronchodilator; *BMI* body mass index; *COPD* Chronic obstructive pulmonary disease; *CAT* COPD Assessment Test; *FEV*_*1*_ forced expiratory volume in 1 s; *FEV*_*1*_*%predicted* forced expiratory volume in 1 s in percent of the predicted value; *GAD* generalized anxiety disorder; *GAD-7* Generalized Anxiety Disorder-7; *GOLD* Global Initiative for Chronic Obstructive Lung Disease; *ICS* inhaled corticosteroid; *IQR* interquartile range; *LABA *long-acting beta_2_-agonist; *LAMA* long-acting muscarinic antagonist; *mMRC* modified Medical Research Council dyspnea scale; *SABA* short-acting beta_2_-agonist; *SD* standard deviation; *SGRQ* St. George’s Respiratory Questionnaire

### Reliability and item analysis

Corrected item–total correlations of the GAD-7 items and Cronbach’s α are summarized in Table 2. Cronbach’s α coefficient for the GAD-7 was 0.869, indicating good internal consistency. All items of the GAD-7 were significantly and positively associated with total GAD-7 scores, with correlations ranging from *r* = 0.461 to *r* = 0.805. Only item 6 (“Becoming easily annoyed or irritable”) caused a slight increased the α coefficient if deleted (Cronbach’s α if deleted = 0.889).

**Table 2:** Corrected item–total correlations and Cronbach’s α if an item was deleted from the GAD-7

	Corrected item–total correlation	Cronbach’s α if item deleted
Item 1	0.750	0.835
Item 2	0.768	0.833
Item 3	0.805	0.828
Item 4	0.685	0.848
Item 5	0.530	0.865
Item 6	0.461	0.889
Item 7	0.634	0.853

*GAD-7* generalized anxiety disorder-7

### Validity analysis

**Fig. 1:**
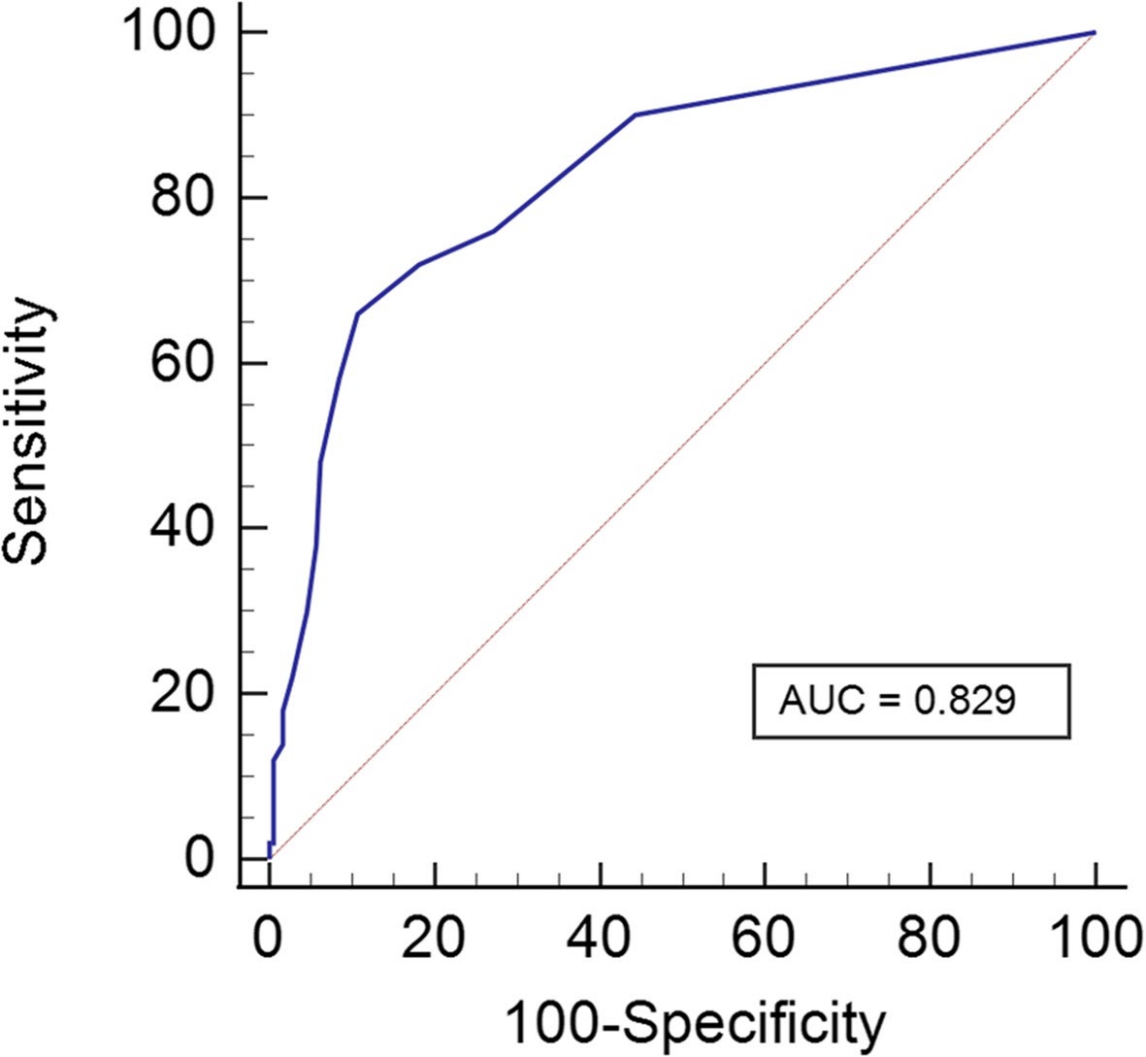
Receiver operating characteristic curve of the GAD-7 for identifying generalized anxiety disorder in patients with chronic obstructive pulmonary disease

ROC curve analysis showed that the GAD-7 had an AUC of 0.829 (95% confidence interval: 0.774–0.876) for identifying GAD (Fig. 1). The optimal cut-off score was achieved at ≥ 4, with the maximum Youden index. The sensitivity, specificity, PPV, NPV, + LR, and—LR were 66.0%, 89.2%, 63.5%, 90.2%, 6.11, and 0.38, respectively (Table 3).

**Table 3:** Screening performance of the GAD-7 for the presence of GAD at various cut-off values

Cut-off value	Sensitivity (%)	Specificity (%)	Youden index (%)	PPV (%)	NPV (%)	+ LR	–LR
2	76.0	72.7	48.7	44.2	91.4	2.79	0.33
3	72.0	81.8	53.8	52.9	91.1	3.96	0.34
4^*^	66.0	89.2	55.2	63.5	90.2	6.11	0.38
5	58.0	91.5	49.5	65.9	88.5	6.81	0.46
6	48.0	93.8	41.8	68.6	86.4	7.68	0.55
7	38.0	94.3	32.3	65.5	84.3	6.69	0.66
8	30.0	95.5	25.5	65.2	82.8	6.60	0.73

*GAD* generalized anxiety disorder; *GAD-7* Generalized Anxiety Disorder-7; *PPV* positive predictive value; *NPV* negative predictive value; *+ LR* positive likelihood ratio; *− LR* negative likelihood ratio

^*^Cut-off score with maximum Youden index

The correlations of the GAD-7 score with health-related quality of life and the symptom burden in COPD were calculated to assess the construct validity (Table 4). Higher GAD-7 scores were significantly associated with higher mMRC scores (*r* = 0.202, *P* = 0.002), CAT scores (*r* = 0.312, *P* < 0.001), and SGRQ total scores (*r* = 0.254, *P* < 0.001). Age, smoking history (pack-years), annual household income, and lung function did not show correlations with GAD-7 scores (Table 4).

**Table 4:** Correlations of GAD-7 score with demographics and clinical factors

Variable	r ^a^	*P* value
Age	0.021	0.752
Smoking pack-years	0.059	0.413
Annual household income	0.041	0.557
Lung function (post-BD)		
FEV_1_, L	− 0.002	0.973
FEV_1_% predicted	− 0.008	0.899
mMRC	0.202	0.002
CAT	0.312	< 0.001
SGRQ		
symptoms	0.193	0.004
activity	0.237	< 0.001
impacts	0.217	0.001
total	0.254	< 0.001

*BD* bronchodilator; *CAT* COPD Assessment Test; *FEV*_*1*_ forced expiratory volume in 1 s; *FEV*_*1*_*%predicted* forced expiratory volume in 1 s in percent of the predicted value; *GAD-7* Generalized Anxiety Disorder-7; *mMRC* modified Medical Research Council dyspnea scale; *SGRQ* St. George’s Respiratory Questionnaire

^a^Spearman’s correlation rho

### GAD-7 performance stratified by demographics and clinical factors

**Fig. 2:**
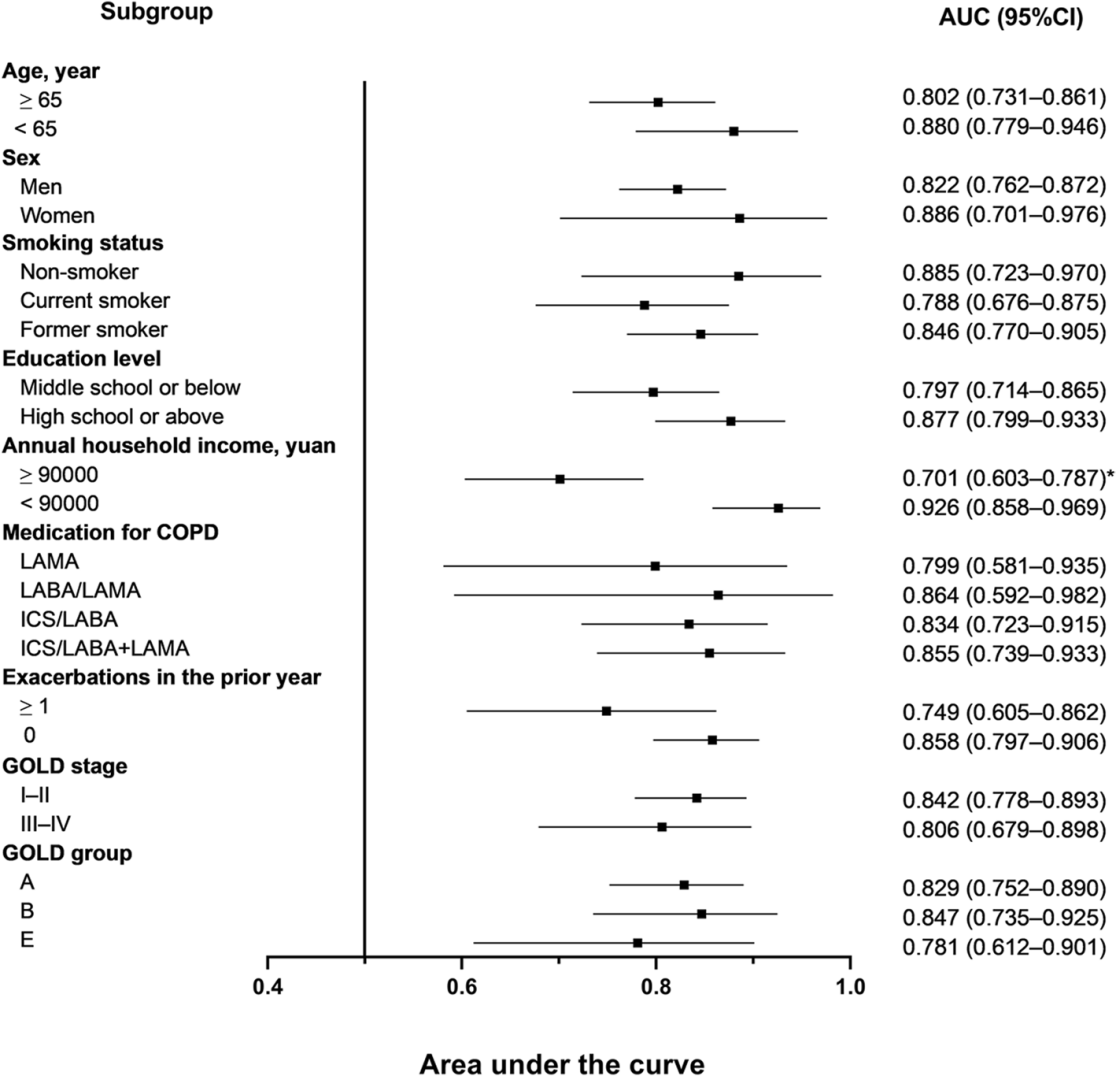
Impact of demographics and clinical factors on the area under the curve (AUC) for the GAD-7; * Significant between-group difference (*P* < 0.05); other between-group differences without this notation were all nonsignificant (*P* > 0.05)

The impacts of demographics and clinical factors on GAD-7 scores are shown in Table 5. GAD-7 scores were significantly higher in patients with COPD with one or more exacerbation in the prior year and were significantly higher in patients classified as GOLD groups B and E. No differences were seen between patients classified by age, sex, smoking status, education level, annual household income, COPD medication use, or GOLD stage. Furthermore, all factors did not have a significant impact on the discriminatory power of the GAD-7 except annual household income, which showed a statistically higher AUC value in a low-income group compared with a high-income group (AUC = 0.926 vs. 0.701, *P* < 0.05, Fig. 2).

**Table 5:** Impact of demographics and clinical factors on GAD-7 scores

	GAD-7 score	*P* value
Age, years		
≥ 65	1.0 (0.0–3.0)	0.567
< 65	1.0 (0.0–4.5)	
Sex		
Men	1.0 (0.0–3.0)	0.071
Women	0.0 (0.0–1.3)	
Smoking status		
Non-smoker	0.0 (0.0–2.5)	0.694
Current smoker	1.0 (0.0–3.0)	
Former smoker	1.0 (0.0–3.0)	
Education level		
Middle school or below	1.0 (0.0–3.0)	0.729
High school or above	1.0 (0.0–4.0)	
Annual household income, yuan		
≥ 90,000	1.0 (0.0–3.0)	0.616
< 90,000	1.0 (0.0–4.0)	
Medication for COPD		
LAMA	1.0 (0.0–5.0)	0.584
LABA/LAMA	3.0 (1.0–4.0)	
ICS/LABA	1.0 (0.0–4.0)	
ICS/LABA + LAMA	1.0 (0.0–3.0)	
Exacerbations in the prior year		
≥ 1	2.0 (0.0–5.5)	**0.011**
0	1.0 (0.0–3.0)	
GOLD stage		
I–II	1.0 (0.0–3.0)	0.294
III–IV	1.0 (0.0–3.5)	
GOLD group		
A	0.0 (0.0–2.0)	**0.001**
B	1.0 (0.0–3.0)	
E	2.5 (0.0–6.0)	

Data are presented as median (IQR). Significant values are presented in bold

*AUC* area under the curve; *COPD* Chronic obstructive pulmonary disease; *GAD-7* Generalized Anxiety Disorder-7; *GOLD* Global Initiative for Chronic Obstructive Lung Disease; *ICS* inhaled corticosteroid; *LABA* long-acting beta_2_-agonist; *LAMA* long-acting muscarinic antagonist

## Discussion

This study was the first to investigate the validity of the Chinese version of the GAD-7 in patients with COPD. We showed that the GAD-7 had good internal consistency and acceptable discriminatory power for detecting GAD in patients with COPD. A cut-off score of ≥ 4 established the best balance between sensitivity and specificity and, thus, presented the optimal cut-off for patients with COPD. The discriminatory power of the GAD-7 did not differ statistically when patients were stratified by COPD severity.

Patients with COPD were more likely to suffer from anxiety than the general population. A meta-analysis reported that the prevalence of GAD in patients with COPD ranged from 6%–33% [23], which is much higher than that reported from epidemiological surveys in the general population (0.2%–6.2%) [24]. Screening questionnaires were useful and time-saving for identifying patients with high risks for developing anxiety disorders. The GAD-7 is a brief screening questionnaire used to assess GAD in primary and specialist care centers. The present study showed that the GAD-7 had an acceptable value regarding measures of consistency in patients with COPD, with a Cronbach’s α coefficient of 0.869. This was consistent with a previous study by Baker et al. [17], which reported a Cronbach’s α coefficient of 0.89 for the GAD-7 in stable patients with COPD in the United States of America.

In the present study, the AUC of the GAD-7 was 0.829, which indicated good discriminatory power. Previous studies have investigated the performance of several anxiety screening questionnaires in patients with COPD. Cheung et al. [25] analyzed the validity of the Hospital Anxiety and Depression Scale-Anxiety subscale (HADS-A) and the Geriatric Anxiety Inventory (GAI) in patients with COPD in New Zealand and found that both scales demonstrated acceptable diagnostic value (AUCs, 0.79 for [HADS-A] and 0.83 for [GAI]). Another study in community-based patients with COPD in Australia reported that the HADS-A and the Beck Anxiety Inventory each had fair discriminatory power for anxiety disorders with AUCs of 0.784 and 0.785, respectively [26]. Compared with the above questionnaires, the GAD-7 is a one-dimensional scale that is more specific for symptoms of GAD. Baker et al. [17] found that the GAD-7 had a slightly higher AUC value than the HADS-A for identifying any anxiety disorder using the MINI as the gold standard. In comparison, the present study used a clinical diagnosis of GAD by an experienced psychiatrist based on DSM-V criteria as the gold standard and confirmed the validity of the Chinese GAD-7 in patients with COPD in China. Further studies are recommended to compare the performance of the Chinese GAD-7 with other anxiety screening tools in patients with COPD in China.

The present study showed that the optimal cut-off score of the GAD-7 was ≥ 4 in patients with COPD, with a sensitivity of 66.0% and a specificity of 89.2%. The optimum cut-off scores of GAD-7 vary with different counties and different populations. The Chinese version of GAD-7 achieved the maximum sensitivity and specificity at cut-off scores ranging from 5 to 7 in various departments in tertiary hospitals [27, 28]. Studies from other countries reported different optimum cut-off scores of GAD-7, such as ≥ 10 in the primary care clinics in the United States [18] and Spain [29], ≥ 12 in the general population in the Netherlands [30], and ≥ 8 in secondary care clinics in Turkey [31]. Linguistic, ethnic, and cultural differences could all account for this discrepancy.

The reason for the lower sensitivity and lower cut-off scores of GAD-7 in patients with COPD in the present study compared with those in the primary care population and general population [18, 32] may be multifactorial. First, COPD is considered a stigmatized chronic condition, and patients with COPD are more likely to anticipate stigma whether it is related to tobacco smoking or COPD itself [33]. The feeling of stigma in patients with COPD was associated with being reluctant to seek help and unwilling to disclose physical or mental symptoms [34]. Second, most patients with COPD are older adults who may experience, view, and report anxiety symptoms differently from younger adults [35]. Older adults were more likely to report somatic symptoms as their primary concern [36] and did not characterize their concerns as “worry” or “excessive fear” [37, 38]. A previous study recommended that the cut-off score for the GAD-7 in the older general population should be lowered to ≥ 5 [15], which is also supported by the present findings. Third, patients with COPD are more likely to have cognitive impairment compared with healthy subjects [39, 40]. Even though we excluded patients with severe cognitive impairment, there are certainly some patients with mild cognitive deficits in the COPD population. The GAD-7 scale uses *several days*, *more than half the days*, and *nearly every day* to reflect the frequency of anxiety symptoms in the prior two weeks, which may be somewhat confusing for some patients with COPD with amnesia or mild cognitive impairment.

We further analyzed the impact of demographics and COPD-related clinical factors on GAD-7 questionnaire performance. Previous studies observed that clinical anxiety and depression were more common in patients in with GOLD stage IV compared with patients in GOLD stages I and II [41, 42], and they were also more common in GOLD group B and D compared with GOLD group A [43, 44]. However, the screening performance of the GAD-7 between GOLD stages or the updated GOLD groups remains unknown. The present results showed that the GAD-7 questionnaire scores were statistically different when stratified by GOLD groups but not by GOLD stages, and the discriminatory power of the GAD-7 did not differ substantially when stratified by either GOLD stages or GOLD groups. These findings indicated that the GAD-7 is a valid screening questionnaire for patients with COPD with varying disease severities. In addition, we showed that the discriminatory power of the GAD-7 was lower in patients with high household income. The reason for this is not clear and requires further investigation; it is probably because patients with high household incomes perceive higher levels of stigma and, thus, tend to suppress psychological impairment in clinical surveys [45].

There were several limitations in the present study. First, this was a single-center study with a relatively small sample size. Future large-scale studies are needed to confirm the present results. Second, we were ethically obligated to exclude patients with severe cognitive impairment who cannot provide informed consent, which may have resulted in a small sampling bias. Third, concurrent validity was not analyzed by comparing the GAD-7 with other standard measurements of anxiety symptom severity such as the Hamilton Anxiety Rating Scale. Nevertheless, the present study had the strength of using DSM-V diagnosis as the gold standard for validating the GAD-7 in a group of stable patients with COPD in China.

## Conclusions

In conclusion, the present study demonstrated that the GAD-7 was a reliable and valid screening tool for patients with COPD in China with varying severity. We recommend a GAD-7 score ≥ 4 as the optimal cut-off score when applied to patients with COPD. These findings should encourage the broad application of the GAD-7 in patients with COPD to improve early detection of comorbid anxiety, promote appropriate intervention, and achieve better prognosis.

## Data Availability

The datasets generated and analyzed during the current study are not publicly available because other studies involving this data are currently in progress, but data are available from the corresponding author on reasonable request.

## Abbreviations

AUC: Area under the curve
CAT: COPD Assessment Test
COPD: Chronic obstructive pulmonary disease
DSM-V: Diagnostic and Statistical Manual of Mental Disorders, 5th edition
FEV_1_: Forced expiratory volume in 1 s
FVC: Forced vital capacity
GAD: Generalized Anxiety Disorder
GAI: Geriatric Anxiety Inventory
GOLD: Global Initiative for Chronic Obstructive Lung Disease
HADS-A: Hospital Anxiety and Depression Scale-Anxiety subscale
LR: Likelihood ratio
MINI: Mini International Neuropsychiatric Interview
mMRC: modified Medical Research Council dyspnea scale
NPV: Negative predictive value
PPV: Positive predictive value
ROC: Receiver operating characteristic
SGRQ: St. George’s Respiratory Questionnaire
